# Robot Learning Method for Human-like Arm Skills Based on the Hybrid Primitive Framework

**DOI:** 10.3390/s24123964

**Published:** 2024-06-19

**Authors:** Jiaxin Li, Hasiaoqier Han, Jinxin Hu, Junwei Lin, Peiyi Li

**Affiliations:** 1 Changchun Institute of Optics, Fine Mechanics and Physics, Chinese Academy of Sciences, Changchun 130033, China; 2 University of Chinese Academy of Sciences, Beijing 100049, China

**Keywords:** dynamic motion primitives, admittance control, stiffness primitives, damping primitives, hybrid primitive framework

## Abstract

This paper addresses the issue of how to endow robots with motion skills, flexibility, and adaptability similar to human arms. It innovatively proposes a hybrid-primitive-frame-based robot skill learning algorithm and utilizes the policy improvement with a path integral algorithm to optimize the parameters of the hybrid primitive framework, enabling robots to possess skills similar to human arms. Firstly, the end of the robot is dynamically modeled using an admittance control model to give the robot flexibility. Secondly, the dynamic movement primitives are employed to model the robot’s motion trajectory. Additionally, novel stiffness primitives and damping primitives are introduced to model the stiffness and damping parameters in the impedance model. The combination of the dynamic movement primitives, stiffness primitives, and damping primitives is called the hybrid primitive framework. Simulated experiments are designed to validate the effectiveness of the hybrid-primitive-frame-based robot skill learning algorithm, including point-to-point motion under external force disturbance and trajectory tracking under variable stiffness conditions.

## 1. Introduction

Collaborative robots are being widely applied, and their tasks and environments are becoming more complex. Robots thus require motion skills, flexibility, and adaptability similar to those of human arms [1,2].

There is a large volume of research on how robots acquire motion skills. The initial motion skills of a robot are acquired through the programming of the teaching pendant [3]. For complex motion trajectories, this method is usually difficult to implement, and the technical requirements of the worker are high. Related technological research has provided traditional robot motion planning algorithms, which comprise mainly the random sampling, artificial potential field, and graph search algorithms [4,5,6]. These algorithms usually require precise modeling of the working environment. A complex working environment greatly reduces the algorithm’s efficiency. With advances in artificial intelligence technology, learning from demonstration (LfD) has gradually become the main way for robots to acquire moving skills [7,8]. Ude et al. recorded the human body teaching trajectory with optical tracking equipment, used a B-spline wavelet to fit the teaching trajectory, and changed the shape of the trajectory by changing the coefficients of the B-spline curve to meet the needs of different tasks [9]. Ye et al. interpolated manipulator trajectory with quintic B-spline curves and achieved multi-objective trajectory optimization that simultaneously optimizes traveling time, energy consumption, and mean jerk [10]. Ijspeert et al. proposed the motion planning of a robot based on a dynamic motion primitive (DMP) algorithm [11]. The DMP algorithm is a demonstration learning algorithm that comprises a second-order system and nonlinear terms. The second-order system ensures that the robot moves to the target point. Through changing the weight of the nonlinear term, the trajectory can have good generalizability. A DMP algorithm based on Gaussian mixture regression has been proposed for a robot to learn multiple teaching trajectories [12]. This article proposed a DMP motion planning algorithm based on task parameters and adjusted the DMP model according to the position of the robot. The parameters of the robot enable man–machine collaborative handling and assembly [13]. Cohen et al. proposed a methodology for learning the manifold of task and DMP parameters, which facilitates runtime adaptation to changes in task requirements while ensuring predictable and robust performance [14]. Paraschos et al. proposed using probabilistic motion primitives (PROMPs) to model the trajectory distribution learned from random movements and Carvalho et al. proposed to combine ProMPs with the Residual Reinforcement Learning (RRL) framework, to account for both, corrections in position and orientation during task execution [15,16]. Wang et al. proposed an improved probabilistic motion primitive algorithm and applied it to the air of a lower-extremity exoskeleton, adopting black-box optimization of the PROMP, so that the exoskeleton adapts to different wearers and improves the adaptive ability of the system [17]. Zhang et al. proposed a new trajectory learning scheme of a limb exoskeleton robot based on dynamic movement primitives (DMPs) combined with reinforcement learning (RL) [18]. To address these challenges, a trajectory learning and modification method based on improved dynamic movement primitives (DMPs), called FDC-DMP, is proposed. The method introduces an improved force-controlled dynamic coupling term (FDCT) that uses virtual force as coupling force. This enhancement enables precise and flexible shape modifications within the target trajectory range [19]. This article addresses a new way of generating compliant trajectories for control using movement primitives to allow physical human–robot interaction where parallel robots (PRs) are involved [20]. Khansari-Zadeh et al. proposed a stable estimator of the dynamical systems algorithm as a demonstration learning algorithm. According to the demonstration trajectory, the probability relationship between the robot’s position and velocity is established, and the Lyapunov stability theory is adopted to ensure that the robot moves to the target. The algorithm has good adaptability to disturbances in time and space [21]. Zhang et al. proposed a stable estimator of dynamical systems algorithm based on neural network optimization. This estimator improves the accuracy of the motion trajectory and handles high-dimensional data [22].

There is a large volume of research on how to improve robot compliance. Most of this research is based on admittance control [23]. As research has deepened, it has been found that the stiffness and damping parameters of the human arm change during a working process [24,25]. Therefore, how to change the parameters of the admittance model according to the work task has become a research hotspot. Yang et al. proposed using a neural network to optimize the parameters of the impedance model, which improves the adaptability of the robot to uncertain environments and ensures the stability of the control system [26]. Zeng et al. proposed an extended TbD system which can also enable learning stiffness regulation strategies from humans and Franklin et al. proposed indirectly estimating the endpoint stiffness of the human arm using electromyography (EMG) during reaching movements [27,28]. By collecting the surface electromechanical signals of the human body in a demonstration task, the change in the stiffness of the human arm during the task can be obtained. The stiffness parameters are applied to the admittance control model, and the robot has the same flexibility as the human arm. Yu, X. et al. adopted demonstration learning to collect the movement trajectory and arm stiffness information of the human arm during the process of operating a drinking fountain and modeled the movement trajectory and stiffness, so that a robot could also press the drinking fountain lever to complete the task of fetching water [29]. Peternel et al. completed a sawing task through human–computer cooperation [30]. Stiffness information of the arm is obtained by detecting the electromyographic signal of the human arm. The stiffness of the robot decreases as the stiffness of the human arm increases. As the stiffness of the human arm decreases, the rigidity of the robot increases to realize a sawing action.

**Figure 1:**
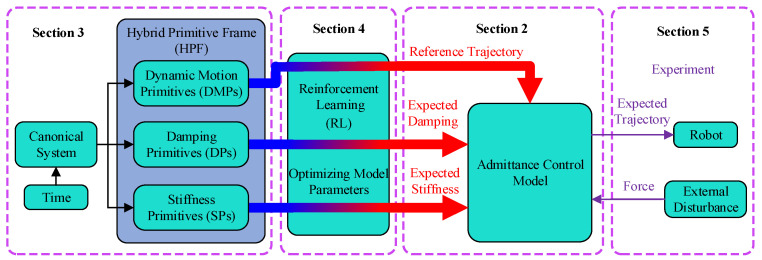
Framework of a robot learning human-like arm.

In this paper, we propose the hybrid primitive framework (HPF) that enables robots to have motion skills and flexibility similar to human arms and optimize the parameters of the hybrid primitive framework using the policy improvement with path integrals (PI2) algorithm to cope with different tasks. Firstly, the end of the robot is dynamically modeled using an admittance control model to give the robot flexibility. Secondly, the robot’s motion trajectory is modeled using dynamic movement primitives (DMPs). Stiffness primitives (SPs) and damping primitives (DPs) are proposed to model the stiffness parameters and damping parameters in the admittance model. The dynamic movement primitives, stiffness primitives, and damping primitives are referred to as the hybrid primitive framework. According to different tasks, the PI2 algorithm is used to iteratively learn the parameters of the hybrid primitive framework to adapt to task requirements, enabling the robot to have adaptability similar to human arms. Finally, simulation experiments are designed under external force disturbance and constant force tracking under variable stiffness conditions to validate the effectiveness of the algorithm. Compared to the previous research, this article innovatively proposes modeling the stiffness and damping parameters in the admittance control model using the stiffness primitives and damping primitives. By coupling these with dynamic movement primitives, the hybrid primitive framework is proposed, enabling the robot to possess both motion skills and compliance skills. The entire framework of this article is shown in Figure 1.

The remainder of the paper is organized as follows. Section 2 presents the admittance control model, Section 3 introduces the HPF, Section 4 describes the optimization of the HPF parameters through the PI2 algorithm, Section 5 reports on simulation experiments based on HPF, and Section 6 draws conclusions from the results of the study.

## 2. Admittance Control Model

To endow robots with compliance, this paper establishes an admittance control model for robot terminals. The admittance model is expressed as

(1) $$f=k(x_{e}-x_{r})+d(v_{e}-v_{r}).$$

where f is the disturbance force; k is the stiffness; d is the damping; $x_{r}$ and $v_{r}$ are, respectively, the reference positions and velocities; and $x_{e}$ and $v_{e}$ are, respectively, the expected positions and velocities. During operation, robots need to move according to the reference trajectory (task trajectory). When subjected to external disturbances, the robot deviates from the reference trajectory. When there is no external force, the robot continues to move along the reference trajectory. The admittance control model is transformed by Laplace transformation to obtain the transfer function:

(2) $$\frac{x(s)}{f(s)}=\frac{1}{ds+k}.$$

It is a first-order system. The interaction force between the robot and the external environment is approximately equivalent to the step signal. The step response of a first-order system is always stable without oscillation, so the admittance control model is stable [31,32].

## 3. Hybrid Primitive Framework

In this paper, we use DMPs to model the motion trajectory. Compared with traditional programming, the use of DMPs is more efficient and stable and has good generalization ability. The DMP framework mainly comprises a stable convergent second-order system and nonlinear forcing terms, which ensure that the robot converges to the target point while moving along a specific trajectory. The DMP model is defined as follows [33,34]:

(3) $$\tau \overset{\cdot \cdot }{x}=\alpha (\beta (p-x)-\overset{\cdot }{x})+f(s).$$

where α and β are predefined constants. In addition, for the second-order system to respond more rapidly and reach the target point, the system needs to satisfy α=4β, which lets the second-order system be in a critically damped state. x, $\overset{\cdot }{x}$, and $\overset{\cdot \cdot }{x}$ are, respectively, the position, velocity, and acceleration of the system. *f*(*s*) is a nonlinear forcing term. Here, *s* is the phase, which monotonically changes from 1 to 0 during movement and satisfies $\tau s=-\alpha_{s}s$. The system is known as a canonical system. $\alpha_{s}$ is a predefined constant that satisfies $e^{-\alpha_{s}}\to 0$. τ is a temporal scaling factor. The nonlinear forcing term is defined as follows:

(4) $$f(s)=\frac{\sum_{i=1}^{N}\omega_{i}\varphi_{i}(s)}{\sum_{i=1}^{N}\varphi_{i}(s)}s(p-x_{0}),\;\varphi_{i}(s)=\exp(-h_{i}{\left(s-c_{i}\right)}^{2}).$$

where $\omega_{i}$ denotes the weights of the Gaussian basis functions; $\varphi_{i}(s)$ denotes the Gaussian basis functions, and each basis function $\varphi_{i}(s)$ is weighted by parameter $\omega_{i}$; $c_{i}$ denotes the centers of the Gaussian basis functions; $h_{i}$ denotes the variances of the Gaussian basis functions; and *N* is the number of Gaussian basis functions. The trajectory shape is changed by changing the weights of the Gaussian basis functions.

In accordance with the definition of the nonlinear forcing term in the DMP model, this paper proposes stiffness primitives (SPs) and damping primitives (DPs) to represent the variation of stiffness parameters and damping parameters in the admittance model. The SP model is defined as follows:

(5) $$k(s)=\frac{\sum_{i=1}^{M}\omega_{i}^{s}\varphi_{i}^{s}(s)}{\sum_{i=1}^{M}\varphi_{i}^{s}(s)},\;\varphi_{i}^{s}(s)=\exp(-h_{i}^{s}{\left(s-c_{i}^{s}\right)}^{2}).$$

where *k*(*s*) consists of *M* Gaussian basis functions $\varphi_{i}^{s}(s)$, and $c_{i}^{s}$ denotes the center of each Gaussian basis function. $h_{i}^{s}$ denotes the variances of each Gaussian basis function. $\omega_{i}^{s}$ denotes the weights of the Gaussian basis functions and determines the shape of the stiffness profiles. DPs have the same form. The DP model is defined as follows:

(6) $$d(s)=\frac{\sum_{i=1}^{Q}\omega_{i}^{d}\varphi_{i}^{d}(s)}{\sum_{i=1}^{Q}\varphi_{i}^{d}(s)},\;\varphi_{i}^{d}(s)=\exp(-h_{i}^{d}{\left(s-c_{i}^{d}\right)}^{2}).$$

This article proposes to refer to DMPs, SPs, and DPs as the hybrid primitive framework (HPF), each of which is driven by system phases to control the motion and compliance of robots together.

## 4. HPF Parameter Optimization Based on the PI2 Algorithm

The weight parameters in the HPF are usually determined through demonstration learning by collecting the motion trajectories of the demonstrator, arm stiffness information, etc. The weight values are solved using the locally weighted regression (LWR) algorithm. This method has the characteristics of fast learning speed and simplicity. However, for motion tasks with complex trajectory and stiffness variations, the demonstrator cannot demonstrate suitable trajectories and stiffness, and the weight parameters of the model in the HPF cannot be determined through demonstration learning. It is important to enable robots to break free from reliance on demonstration learning, possess independent thinking capabilities, and autonomously determine appropriate weight parameters according to tasks. Therefore, this section proposes a weight parameter learning method for HPF based on the policy improvement with path integrals (PI2) [35,36].

Many studies have analyzed the optimization problem of the DMP model based on PI2 and proposed various improved methods [37]. In this paper, this idea is applied to the learning of HPF weight parameters, including the learning of weight parameters in the DMP, SP, and DP components of HPF. The PI2 algorithm implements policy improvement in the form of path integration, which neither requires matrix inversion nor uses gradient descent for parameter optimization, thus avoiding the problem of numerical instability in the iteration process, and this algorithm requires no other free parameters except for exploration noise. The PI2 algorithm belongs to reinforcement learning algorithms. The corresponding cost function is determined based on the task requirements, and after multiple learning iterations, the cost value gradually converges, obtaining the weight parameters of HPF that meet the task requirements.

The DMP model is rewritten as follows:

(7) $$\tau^{2}{\overset{\cdot \cdot }{x}}_{t}=f_{t}+{\left(g_{t}^{m}\right)}^{T}(w_{m}+\varepsilon_{t}^{m}),$$

(8) $$f_{t}=\alpha (\beta (p-x_{t})-\overset{\cdot }{x_{t}}),$$

(9) $$g_{t}^{m}=\frac{s(g-x_{0})}{\sum_{i=1}^{N}\psi_{i}^{m}(s)}\left[\begin{array}{c} \psi_{1}^{m}(s)\\ \vdots \\ \psi_{1}^{m}(s) \end{array}\right],$$

(10) $$w_{m}={\left[\begin{array}{ccc} w_{1}^{m} & \cdots & w_{N}^{m} \end{array}\right]}^{T}.$$

where $w_{m}$ represents the weight parameters in the DMP model, which determine the robot’s motion trajectory, and $\varepsilon_{t}^{m}$ represents the exploration noise vector of the weight parameters in the DMP model in the PI2 algorithm.

The SP model is rewritten as follows:

(11) $$k_{t}={\left(g_{t}^{s}\right)}^{T}(w_{s}+\varepsilon_{t}^{s}),$$

(12) $$g_{t}^{s}=\frac{1}{\sum_{i=1}^{N}\psi_{i}^{s}(s)}\left[\begin{array}{c} \psi_{1}^{s}(s)\\ \vdots \\ \psi_{N}^{s}(s) \end{array}\right],$$

(13) $$w_{s}={\left[\begin{array}{ccc} w_{1}^{s} & ,\cdots , & w_{N}^{s} \end{array}\right]}^{T}.$$

where $w_{s}$ represents the weight parameters in the SP model, determining the variation of stiffness parameters in the admittance control model, and $\varepsilon_{t}^{s}$ represents the exploration noise vector of the weight parameters in the SP model in the PI2 algorithm.

The DP model is rewritten as follows:

(14) $$d_{t}={\left(g_{t}^{d}\right)}^{T}(w_{d}+\varepsilon_{t}^{d}),$$

(15) $$g_{t}^{d}=\frac{1}{\sum_{i=1}^{N}\psi_{i}^{d}(s)}\left[\begin{array}{c} \psi_{1}^{d}(s)\\ \vdots \\ \psi_{N}^{d}(s) \end{array}\right],$$

(16) $$w_{d}={\left[\begin{array}{ccc} w_{1}^{d}, & \cdots & ,w_{N}^{d} \end{array}\right]}^{T}.$$

where $w_{d}$ represents the weight parameters in the DP model, determining the variation of damping parameters in the admittance control model, and $\varepsilon_{t}^{d}$ represents the exploration noise vector of the weight parameters in the DP model in the PI2 algorithm.

In the learning process, the exploration noise vector is added to the HPF weight parameters, and different noise variances $\Sigma_{\varepsilon }$ can be specified, so that the robot can add different levels of noise vectors in the process of learning the trajectory, stiffness, and damping. The process of learning HPF weight parameters is as follows:

Firstly, at each moment, K noise vectors are generated, corresponding to K trajectory curves, stiffness curves, and damping curves, where the cost value of the *k*-th trajectory at time *i* is $J(\tau_{i,k})$, composed of the final cost $\Phi_{t_{n},k}$ of this movement and the immediate cost $r_{t_{n},k}$ of all subsequent time steps:

(17) $$J(\tau_{i,k})=\Phi_{t_{n},k}+\sum_{i=1}^{N-1}r_{t_{i},k}.$$

where *n* is the total number of steps in the motion. The final cost $\Phi_{t_{n},k}$ is determined based on the task requirements. For example, the final cost function $\Phi_{t_{n},k}$ can be defined based on the distance between the robot’s final position and the target position, where a smaller distance indicates a lower cost function value, indicating that the robot can better accomplish the task. The immediate cost $r_{t_{n},k}$ is determined based on the characteristics of the robot’s motion parameters. For example, acceleration parameters can be chosen as components of the immediate cost, where they represent the requirement for the smoothness of the robot’s motion trajectory.

The probability $P(\tau_{i,k})$ of the *k*-th trajectory at time *i*-th is realized through an exponential function and normalization of its corresponding cost value, defined as follows:

(18) $$P(\tau_{i,k})=\frac{\exp(-\frac{1}{\lambda }J(\tau_{i,k}))}{\sum_{k=1}^{K}\exp(-\frac{1}{\lambda }J(\tau_{i,k}))}.$$

A higher cost of the trajectory corresponds to a lower corresponding probability. According to the probability $P(\tau_{i,k})$, it is determined that the *k*-th trajectory explores the noise value weight at the *i*-th moment, and then the K noise values are weighted to obtain the update amount of the weight parameters in the HPF at the *i*-th moment:

(19) $$\delta w_{t_{i}}=\sum_{k=1}^{K}\left[P(\tau_{i,k})M_{t_{i},k}\varepsilon_{t_{i},k}\right].$$

After the update of the weight parameter is calculated for all times, the final update amount for the weight of the *j*-th Gaussian basis function in the HPF during this learning is determined according to the following formula.

(20) $${\left[\delta w\right]}_{j}=\frac{\sum_{i=0}^{N-1}(N-i)\psi_{j,t_{i}}{\left[\delta w_{t_{i}}\right]}_{j}}{\sum_{i=0}^{N-1}\psi_{j,t_{i}}(N-i)}.$$

The learning process of the HPF weight parameters based on the PI2 algorithm is shown in Table 1.

## 5. Experiment

The robot learning method for human-like skills in this paper is applicable to robots with any number of degrees of freedom. This algorithm is used for controlling the end-effector of the robot, and the forward and inverse kinematics algorithms of the robot are not the focus of this study. In the simulation experiments of this section, the structure of the robot is not specifically introduced. The term “robot motion trajectory” refers to the “end-effector motion trajectory,” which can be equivalently treated as a point. The effectiveness of the proposed algorithm is verified by observing the motion trajectory of this point. In this section, we complete simulation experiments based on common robot tasks and analyze the experimental data.

- Task 1: Passing point experiments conducted in a disturbed environment

In the simulation experiment, the robot needs to move from the starting position to the target position. During the movement, the robot is subjected to external disturbance forces and must pass through task points at specified times. Based on the algorithm proposed in this paper, the robot can exhibit human-like compliance while resisting external disturbances, passing through task points, and reaching the target point. The cost function during the learning process can be determined by the following five rules:

Rule 1: The robot reaches the goal point. The speed when reaching the goal point is as low as possible.

Rule 2: The robot passes through the task point.

Rule 3: The robot runs with a low acceleration to ensure smoothness of the trajectory.

Rule 4: The robot has low stiffness while completing the task, such that it has better compliance.

Rule 5: The robot has low damping characteristics that reduce the energy consumption of the system.

According to the above rules, the final cost ϕ of one movement can be determined as follows:

(21) $$\phi =\frac{1}{2}W_{goal}{\left(p_{t\_end}-p_{goal}\right)}^{2}+\frac{1}{2}W_{vel}{\left(\overset{\cdot }{p_{t\_end}}\right)}^{2}.$$

where $W_{goal}$ is the cost weight of the distance between the position and the goal point at the last moment; $p_{t\_end}$ is the position at the last moment; $p_{goal}$ is the goal point; $W_{vel}$ is the cost weight of the speed at the last moment; and $\overset{\cdot }{p_{t\_end}}$ is the velocity at the last moment.

The immediate cost $r_{t}$ is

(22) rt=12WviaQt+12Wacc(pt··)2+12Wstiffkt2+12Wdampdt2Qt= 0 (pt_via−pvia)2 t≠tviat=tvia.

where $W_{via}$ is the cost weight through the task points; $Q_{t}$ is the cost of passing the task point; $p_{via}$ is the task point; $t_{via}$ is the task point time; $p_{t\_via}$ is the position of the robot at time $t_{via}$; $W_{acc}$ is the cost weight of acceleration during motion; $\overset{\cdot \cdot }{p_{t}}$ is the acceleration at time *t*; $W_{stiff}$ is the cost weight of stiffness; $k_{t}$ is the stiffness at time *t*; $W_{damp}$ is the cost weight of damping; and $d_{t}$ is the damping at time *t*.

**Figure 2:**
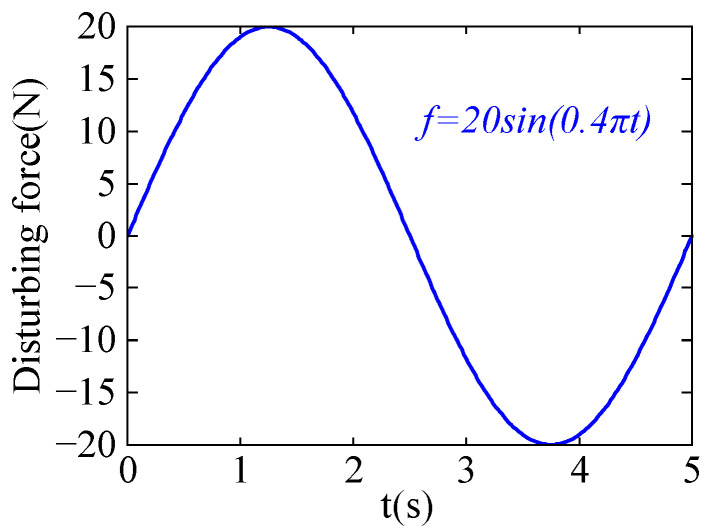
Disturbance force.

In the simulation experiment, the robot’s starting point is (0 m, 0 m), and the target point is (1 m, 1 m), with a motion duration of 5 s. It is required to pass through the task point (0.6 m, 0.8 m) at $t_{via}$ = 2.5 s. To simulate the complex disturbance forces in an actual working environment, sinusoidal disturbance forces are applied in both the x and y directions during the experiment. The disturbance force curve is shown in Figure 2.

**Figure 3:**
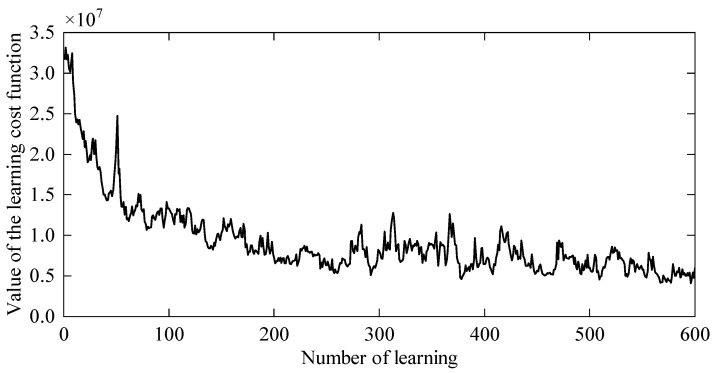
The learning cost value.

The entire learning process iterated 600 times. Since the learning objective is divided into five parts, the learning cost mainly includes point-passing cost, target point cost, acceleration cost, stiffness cost, damping cost, and end velocity cost. As shown in Figure 3, with the increase in the number of iterations, the total learning cost value gradually decreases and converges.

**Figure 4:**
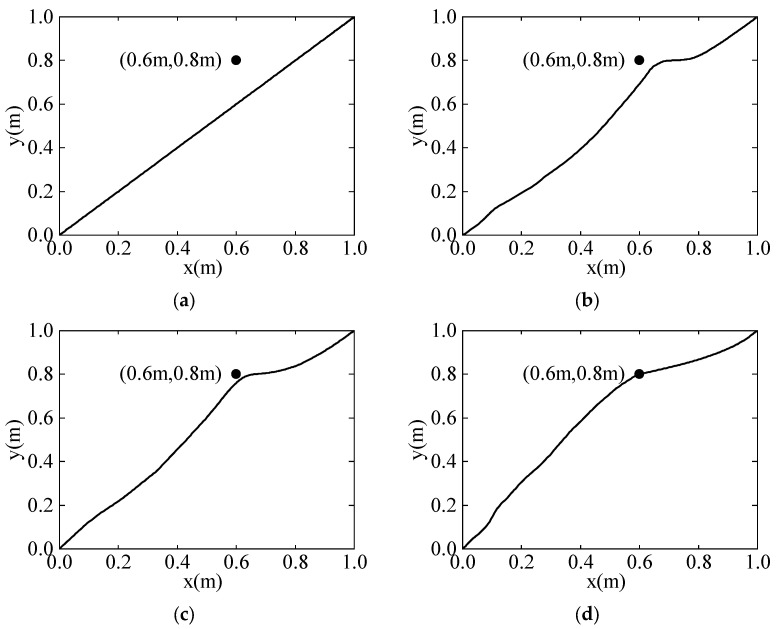
The motion trajectory after learning iterations. (**a**) Initial motion trajectory. (**b**) Motion trajectory after 100 iterations of learning. (**c**) Motion trajectory after 200 iterations of learning. (**d**) Motion trajectory after 600 iterations of learning.

Figure 4 shows the motion trajectories of the robot during the learning process. Figure 4a displays the initial motion trajectory of the robot with the task points. It can be observed that although the robot’s motion trajectory can reach the target point (1 m, 1 m), it deviates from the task point (0.6 m, 0.8 m). Figure 4b displays the motion trajectory of the robot after 100 learning iterations, showing a tendency toward the target point. Figure 4c displays the motion trajectory of the robot after 200 learning iterations; the robot gradually approaches the task point. Figure 4d displays the motion trajectory of the robot after 600 learning iterations, showing that the motion trajectory passes through the task point (0.6 m, 0.8 m) and reaches the target point (1 m, 1 m).

**Figure 5:**
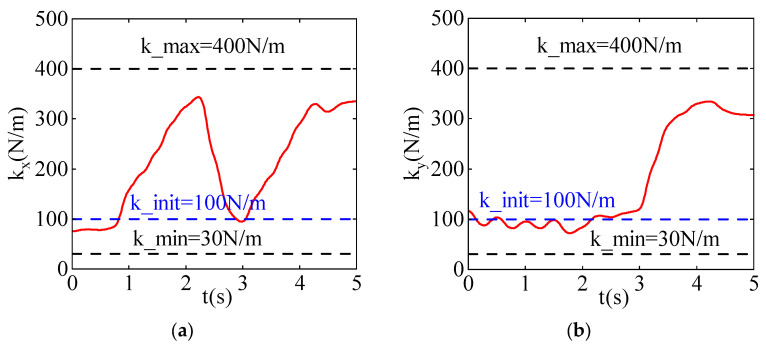
The stiffness curves after learning iterations. (**a**) The stiffness curve in the X-direction. (**b**) The stiffness curve in the Y-direction.

Figure 5 shows the stiffness variation curves in the x and y directions. During the learning process, the maximum stiffness k_max is set to 400 N/m, and the minimum stiffness k_min is set to 30 N/m. The blue curve represents the initial stiffness, the initial stiffness k_init is set to 100 N/m, while the red curve represents the stiffness curve after learning.

Figure 5a illustrates the variation in stiffness in the x-direction. Initially, the stiffness of the robot in the x-direction is low, enhancing the compliance of the robot. This allows the robot to move along disturbance forces smoothly. As the robot gradually approaches the task point, the direction of motion remains aligned with the direction of disturbance forces. To prevent deviation from the task point, stiffness is increased to counteract the disturbance forces and ensure passage through the task point. After passing the task point, the direction of disturbance forces becomes opposite to the direction of robot motion. Therefore, increasing stiffness ensures that the robot can reach the target point.

Figure 5b illustrates the variation in stiffness in the y-direction. In the first half of the time period, the stiffness of the robot in the y-direction is low. This is because the task point in the y-direction is farther from the starting point compared to the x-direction, resulting in consistently lower stiffness in the y-direction to maintain higher compliance. This enables the robot to move along disturbance forces smoothly, ensuring passage through the task point. After passing the task point, the direction of disturbance forces becomes opposite to the direction of robot motion. Therefore, stiffness in the y-direction also needs to be increased to ensure that the robot can reach the target point. 

**Figure 6:**
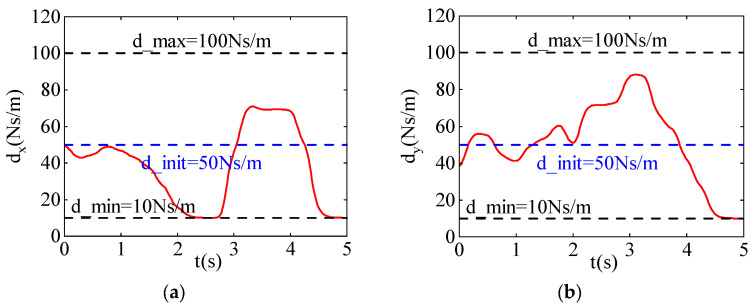
The damping curves after learning iterations. (**a**) The damping curve in the X-direction. (**b**) The damping curve in the Y-direction.

Figure 6 shows the damping variation curves in the x and y directions. During the learning process, the maximum damping d_max is set to 100  Ns/m, and the minimum damping d_min is set to 10  Ns/m. The blue curve represents the initial damping d_init of 50  Ns/m, while the red curve represents the damping curve after learning.

As shown in Figure 6, it can be seen that in the first half of the time period, the damping of the robot in the x and y directions is relatively low. This is because the disturbance force is aligned with the direction of motion, and lower damping allows the robot to better follow the external force. In the second half of the time period, the damping increases significantly because the disturbance force is opposite to the direction of motion. By increasing the damping, the disturbance force is dissipated, preventing the robot from deviating from the target point.

Through analysis, it was found that under the influence of the hybrid primitive, the robot can have motion capabilities, variable stiffness, and variable damping similar to those of a human arm. This allows the robot to complete point-to-point tasks in environments with disturbances.

B. Task 2: Trajectory tracking experiment conducted in a variable stiffness environment

The simulation experiment is set up as follows: the robot moves closely along the surface of a cantilever beam while maintaining a constant contact force, simulating an actual robotic grinding task.

Since the structure and material properties of the cantilever beam are fixed, its deflection curve is also fixed when subjected to a constant force. However, the stiffness of the cantilever beam varies at different positions; the closer to the fixed end, the greater the stiffness. By observing the deviation between the robot’s motion trajectory and the deflection curve of the cantilever beam, a smaller deviation indicates that the robot possesses human-arm-like skills and can complete trajectory-tracking tasks in a variable stiffness environment. The cost function during the learning process can be determined by the following five rules:

Rule 1: The robot reaches the target point. The speed when reaching the target point is as low as possible.

Rule 2: The robot’s motion trajectory is as consistent as possible with the deflection curve of the cantilever beam.

Rule 3: The robot runs with low acceleration to ensure the smoothness of the trajectory.

Rule 4: The robot has low stiffness and thus good compliance while completing the task.

Rule 5: The robot has low damping characteristics that reduce the energy consumption of the system.

According to the above rules, the final cost ϕ of one movement can be determined as follows:

(23) $$\phi =\frac{1}{2}W_{goal}{\left(p_{t\_end}-p_{goal}\right)}^{2}+\frac{1}{2}W_{vel}{\left(\overset{\cdot }{p_{t\_end}}\right)}^{2}.$$

where $W_{goal}$ is the cost weight of the distance between the position and goal point at the last moment; $p_{t\_end}$ is the position at the last moment; $p_{goal}$ is the goal point; $W_{vel}$ is the cost weight of the speed at the last moment; and $\overset{\cdot }{p_{t\_end}}$ is the velocity at the last moment.

The immediate cost $r_{t}$ is

(24) $$r_{t}=\frac{1}{2}W_{gz}{\left(p_{t}-n_{t}\right)}^{2}+\frac{1}{2}W_{acc}{\left(\overset{\cdot \cdot }{p_{t}}\right)}^{2}+\frac{1}{2}W_{stiff}k_{t}^{2}+\frac{1}{2}W_{damp}d_{t}^{2}.$$

where $W_{gz}$ denotes the tracking cost weights for the cantilever beam deflection curves; $p_{t}$ is the position of the robot at time *t*; $n_{t}$ is the deflection of the cantilever beam at time *t*; $W_{acc}$ is the cost weight of acceleration during motion; $\overset{\cdot \cdot }{p_{t}}$ is the acceleration at time *t*; $W_{stiff}$ is the cost weight of stiffness; $k_{t}$ is the stiffness at time *t*; $W_{damp}$ is the cost weight of damping; and $d_{t}$ is the damping at time *t*.

**Figure 7:**
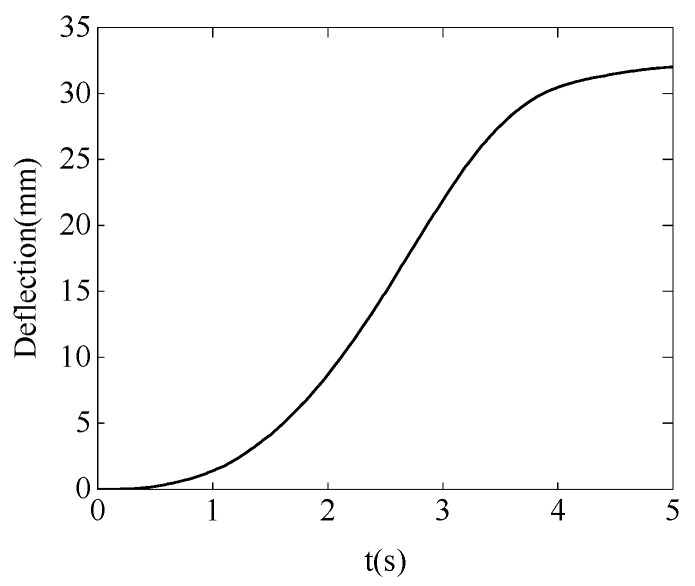
Cantilever beam deflection curve.

In the simulation experiment, the cantilever beam is made of carbon steel with a thickness of 4 mm, a width of 50 mm, and a length of 800 mm. The modulus of elasticity *E* is set to 200 Gpa. According to the deflection curve Equation (25) of the cantilever beam, when subjected to a constant force of 10 N in the y-direction, the deflection curve of the cantilever beam can be obtained, as shown in Figure 7. Therefore, the target position for the robot’s motion is the deflection at the free end of the cantilever beam, which is *y* = 32 mm.

(25) $$y=-\frac{fx^{2}}{6EI}(3l-x),I=\frac{b^{3}h}{12}.$$

where f is the force acting on the surface of the cantilever beam, l is the length of the cantilever beam, E is the modulus of elasticity of the cantilever beam material, and I is the moment of inertia of the cantilever beam section, which is determined by the width b and height h of the cross section.

**Figure 8:**
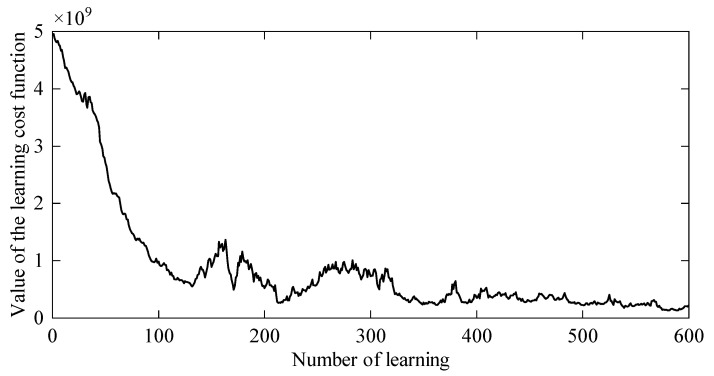
The learning cost value.

The entire learning process iterated 600 times. Since the learning objective is divided into five parts, the learning cost mainly includes tracking cost, acceleration cost, endpoint cost, velocity cost, damping cost, and stiffness cost. As shown in Figure 8, with the increase in the number of iterations, the total learning cost value gradually decreases and converges.

**Figure 9:**
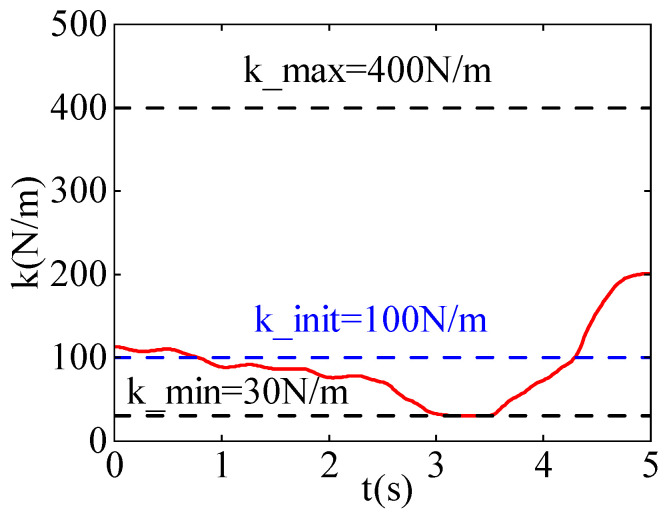
The stiffness curve after learning iterations.

**Figure 10:**
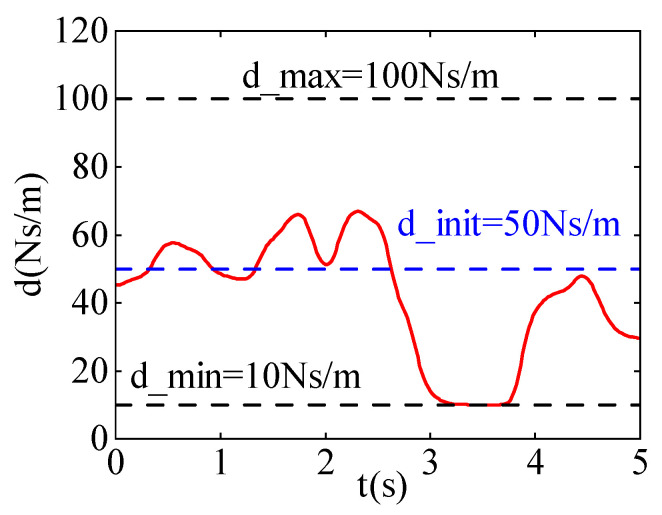
The damping curve after learning iterations.

**Figure 11:**
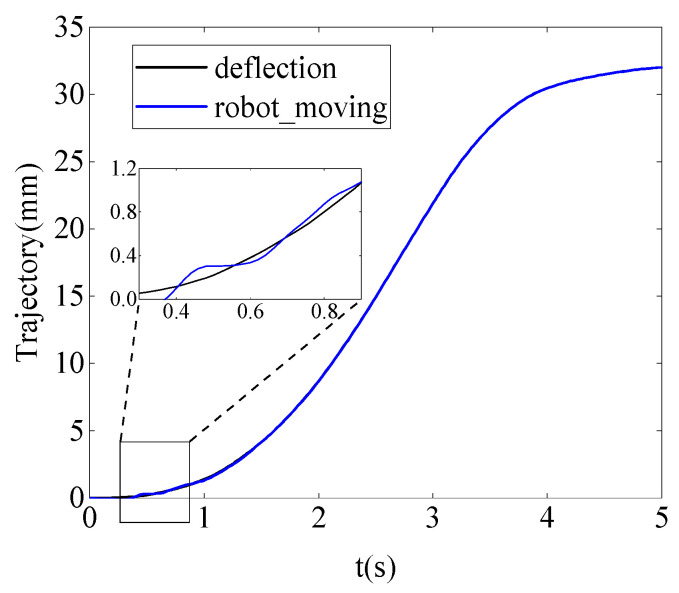
The robot motion trajectory and cantilever beam deflection curve.

**Figure 12:**
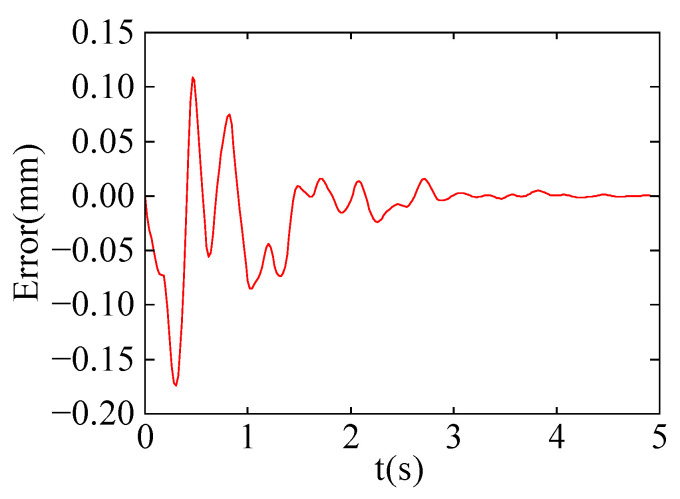
The value deviation between robot motion trajectory and deflection curve.

Figure 9 and Figure 10 show the stiffness and damping variation curves of the robot after learning. In Figure 11, the motion trajectory of the robot after learning is displayed along with the deflection curve of the cantilever beam. The two curves almost overlap, and the error curve between the two trajectories at any time is shown in Figure 12. The error is initially large at the beginning of the motion, then gradually decreases. This is because as the robot moves, the stiffness of the cantilever beam decreases, allowing the robot to better track the deflection curve of the cantilever beam. Throughout the entire motion process, the deviation value remains between ±0.15 mm, indicating that the robot can achieve trajectory tracking under variable stiffness conditions like a human arm, validating the effectiveness of the algorithm proposed in this paper.

## 6. Conclusions

In this paper, a hybrid primitive framework (HPF) was proposed to enable the robot learning of human arm skills. Firstly, the dynamic model of the robot’s end-effector is established using the impedance control theory. Then, the dynamic movement primitive (DMP) algorithm is utilized to model the robot’s motion trajectory. Additionally, stiffness primitives (SPs) and damping primitives (DPs) are introduced to model the stiffness and damping in the impedance model, and the dynamic movement primitive, stiffness primitive, and damping primitive are collectively referred to as the hybrid primitive framework. The policy improvement with path integrals (PI2) algorithm is employed to optimize the parameters of the HPF. Simulation experiments are conducted for point-to-point tasks in a perturbed environment and trajectory tracking in a variable stiffness environment. The experimental results demonstrate that under the influence of the HPF, the robot can learn human-like skills and adapt to complex work tasks and environments, thus verifying the effectiveness of the proposed algorithm in this paper. However, the effectiveness of the hybrid primitive framework was validated through simulation experiments. In the future, the proposed algorithm will be tested with specific robots in practical work scenarios to further verify its effectiveness.

## Figures and Tables

**Table 1:** The learning process of the HPF weight parameters.

Input: (1) Immediate cost function r (2) Final cost function $\Phi_{t_{N}}$ (3) The number of explored noises K (4) Noise variances $\Sigma_{\varepsilon_{m}}$, $\Sigma_{\varepsilon_{s}}$, $\Sigma_{\varepsilon_{d}}$ (5) Initial weight $w_{init}^{m}$, $w_{init}^{s}$, $w_{init}^{d}$ (6) The learning cost function R
Repeat the following steps until the learning cost function R converges:
(1) Calculate the update amount of the weights of each Gaussian basis function in the HPF at each moment. *for i = 1: N* At each moment, the Gaussian basis function weight vector updated in the previous iteration in the HPF is augmented with an exploration noise vector: $\varepsilon_{t_{i}}^{m}$, $\varepsilon_{t_{i}}^{m}$, $\varepsilon_{t_{i}}^{m}$, generating K trajectories, stiffness, and damping curves. *for k = 1: K* $J(\tau_{i,k})=\Phi_{t_{N},k}+\sum_{i=1}^{N-1}r_{t_{i},k}$ $P(\tau_{i,k})=\frac{\exp(-\frac{1}{\lambda }S(\tau_{i,k}))}{\sum_{k=1}^{K}\exp(-\frac{1}{\lambda }S(\tau_{i,k}))}$ $M_{t_{i},k}^{m}=\frac{g_{t_{i},k}^{m}{\left(g_{t_{i},k}^{m}\right)}^{T}}{{\left(g_{t_{i},k}^{m}\right)}^{T}g_{t_{i},k}^{m}}$, $M_{t_{i},k}^{s}=\frac{g_{t_{i},k}^{s}{\left(g_{t_{i},k}^{s}\right)}^{T}}{{\left(g_{t_{i},k}^{s}\right)}^{T}g_{t_{i},k}^{s}}$, $M_{t_{i},k}^{d}=\frac{g_{t_{i},k}^{d}{\left(g_{t_{i},k}^{d}\right)}^{T}}{{\left(g_{t_{i},k}^{d}\right)}^{T}g_{t_{i},k}^{d}}$, $\delta w_{t_{i}}^{m}=\sum_{k=1}^{K}\left[P(\tau_{i,k})M_{t_{i},k}^{m}\varepsilon_{t_{i},k}^{m}\right]$, $\delta w_{t_{i}}^{s}=\sum_{k=1}^{K}\left[P(\tau_{i,k})M_{t_{i},k}^{s}\varepsilon_{t_{i},k}^{s}\right]$, $\delta w_{t_{i}}^{d}=\sum_{k=1}^{K}\left[P(\tau_{i,k})M_{t_{i},k}^{d}\varepsilon_{t_{i},k}^{d}\right]$ *end* *end* (2) Calculate the update amount for the weights of each Gaussian basis function in the HPF at each moment, obtaining the change for the weight of the *j*-th Gaussian basis function in the HPF after each learning iteration. ${\left[\delta w_{m}\right]}_{j}=\frac{\sum_{i=0}^{N-1}(N-i)\psi_{j,t_{i}}^{m}{\left[\delta w_{t_{i}}^{m}\right]}_{j}}{\sum_{i=0}^{N-1}\psi_{j,t_{i}}^{m}(N-i)}$ ${\left[\delta w_{s}\right]}_{j}=\frac{\sum_{i=0}^{N-1}(N-i)\psi_{j,t_{i}}^{s}{\left[\delta w_{t_{i}}^{s}\right]}_{j}}{\sum_{i=0}^{N-1}\psi_{j,t_{i}}^{s}(N-i)}$ ${\left[\delta w_{d}\right]}_{j}=\frac{\sum_{i=0}^{N-1}(N-i)\psi_{j,t_{i}}^{d}{\left[\delta w_{t_{i}}^{d}\right]}_{j}}{\sum_{i=0}^{N-1}\psi_{j,t_{i}}^{d}(N-i)}$ (3) Update the weight parameters of each Gaussian basis function in the HPF after this learning iteration. $w_{m}\leftarrow w_{m}+\delta w_{m}$, $w_{s}\leftarrow w_{s}+\delta w_{s}$, $w_{d}\leftarrow w_{d}+\delta w_{d}$ (4) Based on the updated weights, calculate the learning cost function for this learning iteration. $R=\Phi_{t_{N}}+\sum_{i=1}^{N-1}r_{t_{i}}$
Output: After the learning process, the Gaussian basis function weight parameters in the HPF that satisfy the task requirements are obtained $w_{m}$, $w_{s}$, $w_{d}$.

## Data Availability

The authors confirm that the data supporting the findings of this study are available within the article.

## References

[1] Wang C., Zhao J. (2023). Based on Human-like Variable Admittance Control for Human-Robot Collaborative Motion. Robotica.

[2] Franklin C.S., Dominguez E.G., Fryman J.D., Lewandowski M.L. (2020). Collaborative Robotics: New Era of Human-Robot Cooperation in the Workplace. J. Saf. Res..

[3] Liang K., Wang Y., Pan L., Tang Y., Li J., Lin Y., Pan M. (2024). A Robot Learning from Demonstration Method Based on Neural Network and Teleoperation. Arab. J. Sci. Eng..

[4] Long H., Li G., Zhou F., Chen T. (2023). Cooperative Dynamic Motion Planning for Dual Manipulator Arms Based on RRT*Smart-AD Algorithm. Sensors.

[5] Dai J., Zhang Y., Deng H. (2024). Novel Potential Guided Bidirectional RRT* With Direct Connection Strategy for Path Planning of Redundant Robot Manipulators in Joint Space. IEEE Trans. Ind. Electron..

[6] Li Y., Cheng H. (2023). APP: A* Post-Processing Algorithm for Robots With Bidirectional Shortcut and Path Perturbation. IEEE Robot. Autom. Lett..

[7] Wang K., Fan Y., Sakuma I. (2024). Robot Grasp Planning: A Learning from Demonstration-Based Approach. Sensors.

[8] Hu Y., Abu-Dakka F.J., Chen F., Luo X., Li Z., Knoll A., Ding W. (2024). Fusion Dynamical Systems with Machine Learning in Imitation Learnissssng: A Comprehensive Overview. Inf. Fusion.

[9] Ude A., Atkeson C.G., Riley M. Planning of Joint Trajectories for Humanoid Robots Using B-Spline Wavelets. Proceedings of the Proceedings 2000 ICRA. Millennium Conference. IEEE International Conference on Robotics and Automation. Symposia Proceedings (Cat. No.00CH37065).

[10] Ye J., Hao L., Cheng H. (2024). Multi-Objective Optimal Trajectory Planning for Robot Manipulator Attention to End-Effector Path Limitation. Robotica.

[11] Ijspeert A.J., Nakanishi J., Schaal S. (2002). Movement Imitation with Nonlinear Dynamical Systems in Humanoid Robots. Proceedings of the 2002 IEEE International Conference on Robotics and Automation, Vols I–IV, Proceedings.

[12] Calinon S., Guenter F., Billard A. On Learning, Representing, and Generalizing a Task in a Humanoid Robot. https://webofscience.clarivate.cn/wos/alldb/full-record/WOS:000245109300004.

[13] Rozo L., Calinon S., Caldwell D.G., Jimenez P., Torras C. Learning Physical Collaborative Robot Behaviors From Human Demonstrations. https://webofscience.clarivate.cn/wos/alldb/full-record/WOS:000378528900004.

[14] Cohen Y., Bar-Shira O., Berman S. Motion Adaptation Based on Learning the Manifold of Task and Dynamic Movement Primitive Parameters. https://webofscience.clarivate.cn/wos/alldb/full-record/WOS:000658709600010.

[15] Paraschos A., Daniel C., Peters J., Neumann G. (2018). Using Probabilistic Movement Primitives in Robotics. Auton. Robot..

[16] Carvalho J., Koert D., Daniv M., Peters J. (2022). Adapting Object-Centric Probabilistic Movement Primitives with Residual Reinforcement Learning. Proceedings of the 2022 IEEE-RAS 21ST International Conference on Humanoid Robots (Humanoids).

[17] Wang J., Gao Y., Wu D., Dong W. (2023). Probabilistic Movement Primitive Based Motion Learning for a Lower Limb Exoskeleton with Black-Box Optimization. Front. Inform. Technol. Electron. Eng..

[18] Zhang P., Zhang J. (2022). Motion Generation for Walking Exoskeleton Robot Using Multiple Dynamic Movement Primitives Sequences Combined with Reinforcement Learning. Robotica.

[19] Shen N., Mao J., Li J., Mao Z. (2024). Research on Trajectory Learning and Modification Method Based on Improved Dynamic Movement Primitives. Robot. Comput. -Integr. Manuf..

[20] Escarabajal R.J., Pulloquinga J.L., Valera A., Mata V., Valles M., Castillo-Garcia F.J. (2023). Combined Admittance Control With Type II Singularity Evasion for Parallel Robots Using Dynamic Movement Primitives. IEEE Trans. Robot..

[21] Khansari-Zadeh S.M., Billard A. Learning Stable Nonlinear Dynamical Systems With Gaussian Mixture Models. https://webofscience.clarivate.cn/wos/alldb/full-record/WOS:000295583300009.

[22] Zhang Y., Cheng L., Li H., Cao R. (2022). Learning Accurate and Stable Point-to-Point Motions: A Dynamic System Approach. IEEE Robot. Autom. Lett..

[23] Hogan N. (1985). Impedance Control: An Approach to Manipulation: Part II—Implementation. J. Dyn. Syst. Meas. Control-Trans. ASME.

[24] Kim H., Yang W. (2021). Variable Admittance Control Based on Human-Robot Collaboration Observer Using Frequency Analysis for Sensitive and Safe Interaction. Sensors.

[25] Du Z., Wang W., Yan Z., Dong W., Wang W. (2017). Variable Admittance Control Based on Fuzzy Reinforcement Learning for Minimally Invasive Surgery Manipulator. Sensors.

[26] Yang C., Peng G., Li Y., Cui R., Cheng L., Li Z. (2019). Neural Networks Enhanced Adaptive Admittance Control of Optimized Robot-Environment Interaction. IEEE Trans. Cybern..

[27] Zeng C., Yang C., Chen Z., Dai S.-L. (2018). Robot Learning Human Stiffness Regulation for Hybrid Manufacture. Assem. Autom..

[28] Franklin D.W., Leung F., Kawato M., Milner T.E. (2003). Estimation of Multijoint Limb Stiffness from EMG during Reaching Movements. Proceedings of the IEEE EMBS APBME 2003.

[29] Yu X., Liu P., He W., Liu Y., Chen Q., Ding L. (2022). Human-Robot Variable Impedance Skills Transfer Learning Based on Dynamic Movement Primitives. IEEE Robot. Autom. Lett..

[30] Peternel L., Tsagarakis N., Ajoudani A. Towards Multi-Modal Intention Interfaces for Human-Robot Co-Manipulation. https://webofscience.clarivate.cn/wos/alldb/full-record/WOS:000391921702124.

[31] Zhao K., Liu J., Lv X. (2024). A Unified Approach to Solvability and Stability of Multipoint BVPs for Langevin and Sturm-Liouville Equations with CH-Fractional Derivatives and Impulses via Coincidence Theory. Fractal Fract..

[32] Zhao K. (2024). Study on the Stability and Its Simulation Algorithm of a Nonlinear Impulsive ABC-Fractional Coupled System with a Laplacian Operator via F-Contractive Mapping. Adv. Contin. Discret. Models.

[33] Hogan N., Sternad D. (2013). Dynamic Primitives in the Control of Locomotion. Front. Comput. Neurosci..

[34] Wensing P.M., Slotine J.-J. (2017). Sparse Control for Dynamic Movement Primitives. Proceedings of the IFAC PAPERSONLINE.

[35] Liu L., Guo X., Fang Y. (2021). Goal-Driven Motion Control of Snake Robots with Onboard Cameras via Policy Improvement with Path Integrals. Proceedings of the 2021 IEEE International Conference on Robotics and Biomimetics (IEEE-ROBIO 2021).

[36] Lefebvre T., Crevecoeur G. (2019). Path Integral Policy Improvement with Differential Dynamic Programming. Proceedings of the 2019 IEEE/ASME International Conference on Advanced Intelligent Mechatronics (AIM).

[37] Li A., Liu Z., Wang W., Zhu M., Li Y., Huo Q., Dai M. (2021). Reinforcement Learning with Dynamic Movement Primitives for Obstacle Avoidance. Appl. Sci..

